# Contribution to the Personalized Management of the Nosocomial Infections: A New Paradigm Regarding the Influence of the Community Microbial Environment on the Incidence of the Healthcare-Associated Infections (HAI) in Emergency Hospital Surgical Departments

**DOI:** 10.3390/jpm13020210

**Published:** 2023-01-25

**Authors:** Maria-Cristina Mateescu, Simona Grigorescu, Bogdan Socea, Vlad Bloanca, Ovidiu-Dan Grigorescu

**Affiliations:** 1 Faculty of Medicine, Transilvania University of Brasov, 500036 Brasov, Romania; 2 Department of Surgery, University of Medicine and Pharmacy “Carol Davila”, 050474 Bucharest, Romania; 3 Department of Surgery, Victor Babeș University of Medicine and Pharmacy, 300041 Timisoara, Romania

**Keywords:** hospital-acquired infection (HAI), personalized management, community microbial environment, new paradigm

## Abstract

**Background:** The management of acute surgical pathology implies not only the diagnosis–treatment sequence but also an important preventive component. In the surgical hospital department, wound infection is one of the most frequent complications which must be managed both in a preventive and a personalized manner. To achieve this goal, several factors of negative local evolution, contributing to the slowdown of the healing processes, such as the colonization and contamination of the wounds, need to be emphasized and controlled from the first moment. In this context, knowing the bacteriological status at admission ensures the distinction between the colonization and infection processes and could help to manage in an efficient way the fight against bacterial pathogen infections from the beginning. **Methods:** A prospective study was performed for 21 months on 973 patients hospitalized as emergencies in the Plastic and Reconstructive Surgery Department within the Emergency University County Hospital of Brasov, Romania. We analyzed the bacteriological profile of the patients from admission to discharge and the bidirectional and cyclic microorganism dynamics both in the hospital and the community microbial environment. **Results:** Of the 973 samples collected at admission, 702 were positive, with 17 bacterial species and one fungal, with a predominance of Gram-positive cocci at 74,85%. The most frequently isolated strains were Staphylococcus species (86.51% of the Gram-positive/64.7% of the total isolated strains), while Klebsiella at 8.16% and Pseudomonas aeruginosa species at 5.63% were mainly emphasized in the case of Gram-negative bacilli. Two to seven pathogens were introduced after admission, suggesting that the community microbial environment is in a process of evolution and enrichment with hospital pathogens. **Conclusions:** The high level of positive bacteriological samples and the complex associations of the pathogens found at the admission bacteriological screening sustain the new idea that the pathogenic microorganisms existing in the community microbial environment have started to increasingly influence the hospital microbial environment, in contrast with the previous consideration, which emphasized only the unidirectional relationship between hospital infections and the changing bacteriological characteristics of the community environment. This modified paradigm must become the basis of a new personalized approach to the management of nosocomial infections.

## 1. Introduction

In surgical wards, and especially in those where patients with acute pathological entities are treated, Healthcare-Associated Infection (HAI) remains one of the major potential complications that can generate an increase in the morbidity, mortality, and hospitalization period. Unfortunately, in the last two to three years, COVID-19 infection became the central point of global medical interest due to the enormous number of affected subjects, and the average number of hospital admissions in the majority of specialties during the pandemic period was significantly diminished. However, in surgical departments (especially in plastic, reconstructive, and burn departments), HAI remained a serious burden on the safety of the patients.

Regarding the plastic surgery specialty, although most of the elective surgeries were postponed (mainly in 2020 and 2021), trauma, infections, and burn cases continued to maintain at least at the same level of presentation in the emergency department. For this reason, the number of cases that required admission and the subsequent need for intervention remained similar to the period immediately preceding the pandemic [1,2,3,4,5].

According to a recent WHO analysis, the prevalence of HAI has had a growing trend. In acute care hospitals in low/middle income countries, 15 out of every 100 patients will acquire at least one HAI and, furthermore, one of in every 10 of these patients will die. In these countries, of all the types of pathologies complicated with HAI, the surgical cases were characterized by high risk of this kind of association. In Europe, every year, in the acute and long-term care facilities, HAI’s prevalence is at around 8.9 million cases, probably due to the increased number of admissions [6].

In this context, the need for adequate and prompt antimicrobial therapeutic management characterized by an important preventive component is justified by the fact that in the case of patients with complicated infection traumas, the local and general evolution depends on many factors, among which the “initial trauma–gateway–infection” association plays a very important role.

Until now, most research questioned only the etiopathogenetic value of the “gateway (generated by a traumatic agent)–infection” relation, which could be considered a functional binomial.

In our opinion, this bivalent relation is not complete from the functional pathogenetic point of view. We suggest a new paradigm, one of a trivalent relation, which questions (maybe for the first time) the notion of “**causality trinomial: 1. initial trauma (as a known or unknown etiologic factor)—2. (known or unknown) gateway—3. (known or unknown) infection**”, which could be considerate complete. This new notion is based on the fact that the etiological agent, which generates the cutaneous gateway, determines not only the way by which the microorganisms could infect the subject but, importantly, the tissue lesions (more or less extended), which become factors favoring the infection, more so as the contusion/crush mechanism is frequently associated with local circulatory disorders, stimulating the occurrence of the conditions for microorganism development.

This type of infection can be considered a “natural” one, unlike the infection occurring in wounds following contamination with germs from the hospital during hospitalization, considered currently to be a Healthcare-Associated Infection (HAI).

This is why the emphasis on the colonization or infection of each patient’s wounds, even from the start of the hospitalization, has an important role in the correct predictive and personalized assessment of HAI in surgical wards. The problem of nosocomial infections associated with the subsequent occurrence of antibiotic resistance has been the subject of many studies; however, a systematic follow-up in each case of the bacteriological status of patients with acute pathology at hospitalization in a surgical ward has not been included.

Starting from the concept that the colonization of wounds and adjacent integument is one of the important risk factors for potential infection and the deceleration of the healing process of local lesions, we aimed to identify and analyze the bacteriological profile of patients with potential infection evolution from the start of their hospitalization and possible modifications that occur during hospitalization, as a strategy for preventive, predictive, and personalized management (PPPM) implementation [7,8].

The HAI approach in light of the PPPM concept has specifically been encountered until now in terms of analysis and efficient therapy, although, in our opinion, it can contribute efficiently to fighting against the generating factors of the negative evolution mentioned above.

## 2. Working Hypothesis

The introduction of a specific protocol for monitoring nosocomial infections used for the identification and analysis of the characteristic elements of HAIs in the Plastic and Reconstructive Surgery Department within the Emergency University Hospital of Brasov County (EUHBC) led shortly from its introduction to an emphasis on an important number of positive bacteriological results at the start of hospitalization, which confirmed the existence of infections and/or the colonization of patients’ wounds, even from the point of admission into the hospital. These data could be the basis of a correct assessment of the real level of the nosocomial infections from a conceptual point of view, starting from the possibility of its international definition (which requires, among other criteria, the mandatory information on the bacteriological constellation of each patient upon admission to a medical unit).

This research is evidence-based and outlines the main objective of the study in light of the three important directions of current medicine: preventive, predictive, and personalized, part of the concept whose acronym is known in the scientific literature as PPPM.

➢ The **preventive** component, in this case, is represented by:
  ○ The prevention of HAI.
  ○ The prevention of the spread and maximal limitation of bacterial multiresistance in the hospital wards.
  ○ The prevention of antibiotic resistance.

➢ The **predictive** component is represented by:
  ○ Knowing the germs usually found in each ward area (bacteriological map), which leads to the possibility of establishing dedicated antibiotic treatment (based on the statistical bacteriologic results of the ward, which are significantly higher than the initial three-day period of “broad spectrum” treatment);
  ○ Ensuring the supply of the ward with antibiotics from the antibiotic resistance spectrum specific to the most frequent germs found in patients in the ward.
  ○ Anticipating the type of infectious evolution from wound infections.

➢ The **personalized** component is characterized by:
  ○ Individual analysis of the local bacteriological status from the first moment of each patient’s hospitalization.
  ○ Establishing treatment depending on the antibiotic sensitivity test results.
  ○ Ensuring the specific environmental conditions that limit cross-infection with multiresistant germs.

## 3. Materials and Methods

The prospective study was carried out for a period of 21 months (October 2020 to June 2022) on a sample of 973 patients hospitalized for an emergency in the Plastic and Reconstructive Surgery Department within the Emergency University Hospital of Brasov County (EUHBC).

The objective of the study was to analyze the dynamics of the pathogenic microorganisms (especially multiresistant ones) between the hospital and community environment and to show that the evolution mechanism has changed from unidirectional (in the past) to a vicious circle (in the present). It aimed to confirm the higher influence of pathogenic microorganisms from the community environment on the hospital environment, different to what has been considered until recently. The objective was met by the following actions:

1. Analysis of the pathogenic microorganisms at the level of patient wounds from the moment of admission into the hospital (after the interaction between the macroorganisms and microorganisms in the community environment, after the occurrence of the pathological gateway);
2. Analysis of the pathogenic multiresistant microorganisms at the level of wounds from admission, thus entering the hospital environment.
3. Identification of all categories of pathogenic microorganisms frequently existing in the studied in-hospital environment.
4. Identification of pathogenic species entering the community along with the discharge of the studied subjects.

Three inclusion criteria were used: (a) patients with an infectious evolution potential pathology; (b) informed consent of the patients to the study; (c) existence of at least one correct bacteriological examination carried out within the first 48 h of admission.

The samples were collected from the wounds, before primary surgical processing and initiation of the general antibiotic treatment, in compliance with the collection conditions (strict asepsis norms, sterile disposable recipients), and immediately transported to the hospital’s microbiology laboratory.

In order to perform the identification of bacterial stains from the clinical samples, standard microbiological methods were used and in some selected cases were completed by automated methods using the VYTEK 2 system. Regarding the susceptibility of the stains isolated to different antibiotics, for each sample two different methods were used, represented by both diffusometry and microdilution techniques.

The analyzed parameters of the study subjects were: (1) demographic data (age, sex); (2) hospitalization diagnosis; (3) hospitalization period; and (4) the results of bacteriological examinations. The data were collected from the medical charts of patients hospitalized during the study and subsequently recorded in the database and analyzed using statistical processing programs.

## 4. Results

1. The demographic data were the following: 76.77% men and 48.42% women. The average age was 47.76 (min. 12 max. 95).
2. In total, 852 patients hospitalized for initial traumatic pathology and 121 patients with initial sepsis pathology were investigated during the study. The distribution of the hospitalization diagnoses is detailed in Table 1 and Table 2.

**Table 1:** Distribution of hospitalization diagnoses for the entire studied pathology.

DIAGNOSTIC	CASE NO.	PERCENTAGE DISTRIBUTION
**Multiple/mixed** **injuries**	719	73.90%
**Burns**	133	13.67%
**Bone, joint and soft tissue infections**	121	12.44%
	973	100.00%

**Table 2:** Distribution of hospitalization diagnoses for wounds with mixed lesions.

LOCATION OF INJURY	TYPE OF INJURY	CASE NO.	PERCENTAGE
			DISTRIBUTION
	**Musculotendinous and neurovascular**	602	61.87%
**Limbs**			
	Bone and joint	42	4.32%
**Other**		75	7.71%
			**73.90%**

3. The average hospitalization period was eight and twelve days (min. 1, max. 101).
4. The highest incidence of microorganisms found at the time of patients’ hospitalization was noticed in the case of complex (musculotendinous and neurovascular) traumas, followed by traumatic amputations, and infections of soft tissues, bones, joints, and burns (Table 3).

**Table 3:** Correlation between diagnosis and germs at admission.

	Gram Stain	Musculo Tendinous and Neuro Vascular	Traumatic Amputation	Bone, Joint and Soft Tissue Infections	Burns	Crush Injuries	Other Injuries	Bone and Joint Trauma	Total	
**CoNS**	+	113	61	42	47	37	29	15	344	
***S. aureus* (MSSA)**	+	50	16	31	26	15	8	8	154	
***Staphylococcus* spp.**	+	47	28	7	13	17	15	5	132	
***Klebsiella* spp.**	−	26	9	15	6	10	8	5	79	
***Enterococcus* spp.**	+	27	11	7	14	9	9	1	78	
***P. aeruginosa***	−	23	9	4	7	9	5	1	58	
***E. coli***	−	13	4	4	6	5	5	2	39	
***Acinetobacter* spp.**	−	12	1	6	5	5	5	4	38	
***S. aureus* (MRSA)**	+	9	2	7	9	3	3	4	37	
***Proteus* spp.**	−	6	5	9	4	0	6	0	30	
***Streptococcus* spp.**	+	7	2	8	1	2	2	1	23	
***Klebsiella* spp. (ESBL)**	−	2	1	0	1	0	0	1	5	
***Enterobacter* spp.**	+	1	1	1	1	0	0	0	4	
***Enterococcus* spp. (HLAR)**	+	0	0	2	1	0	0	0	3	
***E. coli* ( ESBL)**	−	0	0	2	0	0	0	1	3	
***Serratia* spp.**	−	1	1	0	0	0	0	0	2	
***Serratia* spp. (ESBL)**	−	0	0	0	0	1	0	0	1	
***Candida* spp.**		1	0	0	0	0	0	0	1	
		338	151	145	141	113	95	48	1031	**Total**
		32.78%	14.65%	14.06%	13.68%	10.96%	9.21%	4.66%		**Percentage distribution**

5. Out of the total number of 973 samples collected at hospitalization, 702 (72.15%) were positive (monobacterial and multibacterial), with 1030 bacterial strains (of which there were 771 Gram-positive cocci at 74.85% and 259 Gram-negative bacilli at 25.15%) and one fungal strain isolated. Of the isolated Gram-positive bacteria, the most frequent were Staphylococcus species (86.51% of Gram-positive), representing 64.7% of the total isolated strains, while Klebsiella was 8.16% and Pseudomonas aeruginosa species was 5.63%, mainly emphasized in the case of Gram-negative bacilli (Table 4).

**Table 4:** Distribution of frequently identified microorganisms depending on Gram stain.

			Percentage Distribution
		***STAPHYLOCOCCUS* SPP.**	
**GRAM +**	771	667	64.76%
		*KLEBSIELLA* SPP.	
		84	8.16%
**GRAM −**	259		
		*P. AERUGINOSA*	5.63%
		58	
**Total strains**	1030

6. In total, seventeen bacterial species and one fungal species were identified at the start of hospitalization, which is an important number of microorganisms. Their distribution is presented in Table 5.

**Table 5:** Distribution of isolated microorganisms at the time of hospital admission.

Microorganisms	No.	Percentage Distribution
**CoNS**	344	33.37%
***S. aureus* (MSSA)**	154	14.94%
***Staphylococcus* spp.**	132	12.80%
***Klebsiella* spp.**	79	7.66%
***Enterococcus* spp.**	78	7.57%
***P. aeruginosa***	58	5.63%
***S. aureus* (MRSA)**	37	3.59%
***E. coli***	39	3.78%
***Acinetobacter* spp.**	38	3.69%
***Proteus* spp.**	30	2.91%
***Streptococcus* spp.**	23	2.23%
***Klebsiella* spp. (ESBL)**	5	0.48%
***Enterobacter* spp.**	4	0.39%
***Enterococcus* spp. (HLAR)**	3	0.29%
***E. coli* (ESBL)**	3	0.29%
***Serratia* spp.**	2	0.19%
***Serratia* spp. (ESBL)**	1	0.10%
***Candida* spp.**	1	0.10%
	1031	100.00%

7. We aimed to identify the microbial associations at the start of the hospitalization of patients. The analysis of the bacteriological results revealed that, of the 973 samples collected, 472 were mono-microbial (48.50%), 230 polymicrobial (23.63%), and the remaining 271 were negative samples (27.85%). The microbial associations involved: 159 cases with two germs, 50 cases with three germs, 17 cases with four germs, three cases with five germs, and one case with seven germs.

The presence of a minimum number of ten emphases for bi-microbial, three for tri-microbial, and two for quadri-microbial associations was considered relevant for the study in the analysis of microbial associations. The most frequent types of microbial associations out of the total identified are presented in Table 6, Table 7, and Table 8.

8. Although the results of the microbial associations for hospitalized patients seem “attractive” from a scientific point of view, we considered that the analysis of some types of the multiresistant germs identified was important. Of the total multiresistant species isolated, 49 species drew attention by their higher possibility of infection evolution, out of which *S. aureus* strains (MRSA) at 3.59% were the most frequent, followed by the strains producing extended-spectrum beta-lactamases: *Klebsiella* spp. at 0.48%, *E. coli* at 0.29%, Serratia at 0.10%, and *Enterococcus* spp. (HLAR) at 0.29%. The antibiotic resistance of these strains is presented in Table 9 and Table 10, according to the criterion of decreasing multiresistance. The existence of strains resistant to all the tested antibiotics was noticed for *S. aureus* (MRSA), except for Chloramphenicol, and 11 strains were characterized by a common resistance pattern to Penicillin, Oxacillin, Erythromycin, and Clindamycin, but they were sensitive to the other tested antibiotics. Identical resistance patterns were not identified with respect to the Klebsiella, *E. coli*, and Serratia species.

9. To obtain a more specific image of the bacteriological conglomerate in the studied patients, their bacteriological status was analyzed during hospitalization, including the hospitalization situation (Table 11). The data from Table 11, column 2 (intermediate bacteriological examination) and column 3 (final bacteriological examination) represent the new microorganisms compared to the previous bacteriological examinations.

The data from the above table resulted from the fact that, out of the total number of patients, we considered that new bacteriological samples needed to be collected from 325 patients. Thus, samples were collected for the intermediate bacteriological examination from 56 patients, the intermediate and final bacteriological examination for 160 patients, and the final bacteriological examination from 109 patients. In this context, the results of the collected samples were as follows:

(a) Intermediate bacteriological examination: 157 positive samples (22 initially negative, 91 samples with different germs, 44 samples with the same germs) and 59 negative samples (18 initially negative, 41 initially positive). Out of the newly emphasized microorganisms, only two species different from the community ones were isolated, namely: one *Proteus* spp. (ESBL) strain and one Chryseobacterium indologenes strain.
(b) Final bacteriological examination: 157 positive cultures (19 initially negative, 69 samples with different germs, 69 samples with the same germs) and 112 negative cultures (37 initially negative, 75 initially positive).

## 5. Discussion

The discussion of the obtained results must start from the fact that antibiotic resistance is currently an extremely concerning issue for human civilization. The alarming ascending dynamics of bacterial resistance to many classes of antibiotics led and continues to lead to infections with multiresistant germs and, implicitly, to therapeutic difficulties [5,9,10].

Thus, the interest of this study in emphasizing the inputs and outputs from and within the extra-hospital community component is explainable, represented by germ-bearing patients, especially multiresistant ones, whether they represent the clinical porting component (as infections) or asymptomatic bearers.

Although it would seem paradoxical in the current Romanian context, we believe that dynamic control in the community of the microorganisms with antibiotic resistance is more important than the correct treatment of (wound) infections. Regarding the term ‘community’, we propose that it be understood as an entity having a wider external component (which can be metaphorically called the “civil component”) and a narrower internal one, represented by the hospital environment (also called, metaphorically, the “military component”).

Antibiotic resistance is closely related to the quality of the antibiotic therapy, which in turn implies, first of all, diagnosis of the infection. In the medical–surgical practice and especially in the pathology with sepsis potential (referred to in this study), the infection diagnosis is established based on clinical and paraclinical investigations (microbiological, imaging, and hematological) and indicators. These indicators are useful both for certifying the presence of infection (under its forms: local, regional, and general) but also for its differentiation from wound colonization and contamination status [2].

As not all wounds evolve with infection, only the correct recognition of this entity (by a precise differentiation of the infection from contamination or colonization status) will allow the clinician to choose the local and/or general adequate therapy. Thus, in the case of bacterial colonization and contamination, the therapeutic attitude targets **only** the use of local (topical) antiseptics when changing bandages and **only** exceptionally antibiotic prophylaxis, with specific surgical treatment. Unlike this approach, in the cases in which the presence of infection is certified (under its clinical forms), therapy with antibiotics (with topical, oral, and intravenous administration) is also associated with surgical treatment. The differentiation of the body’s reaction to the action of microorganisms and the classification in one of the three major classes (colonization–contamination–infection) is based on the previously mentioned clinical and paraclinical indicators [2,3,8].

This study was primarily based on laboratory results. These were only obtained after clinical examination raised the suspicion of infection in the analyzed cases. Based on this consideration, of the 973 subjects, 56 subjects were subjected to intermediate bacteriological examination, with 160 patients subjected to intermediate and final bacteriological examinations and 109 subjects to bacteriological examination at discharge. In total, 648 subjects (including 212 with negative samples at hospitalization) were not subjected to the intermediate and/or final bacteriological examination due to the absence of local and general signs of infection, considered a colonization status and not a real infection. The short hospitalization period of these patients (average period 4, 64 days) was also an additional argument that emphasized their evolution without infection.

The undamaged integument is normally impenetrable for most germs, with the role of controlling the microbial populations from the skin flora and preventing the colonization and contamination of the underlying structures with potentially pathogenic germs from functional and microbiological points of view. The interruption of cutaneous continuity and integrity, after a trauma (generated by various mechanisms), leads to the possibility of inoculating microorganisms from the skin microbacterial flora (in addition to those associated with the etiologic agent), thus creating a favorable environment for the colonization and/or contamination of tissues from the profound layers. After these processes, there is the possibility of evolution with infection in the presence of factors related to the microorganism (dose, virulence, and pathogenicity) and macroorganism (immune status, peripheral circulation, etc.) [4,8,11,12].

In relation to these data, in the case of the patients included in this study, characterized by post-traumatic pathology and potential septic risk, it must be taken into account that there are several local particular factors that make the patients more susceptible to the contamination of wounds and the subsequent potential evolution to infection, of which we mention: the retention of foreign bodies, devitalization of tissues, decrease in local circulation, and the alteration in local defense mechanisms [13]. Of the 973 samples collected at hospitalization, 702 (72.15%) were positive, which explains the high risk of infection evolution and, therefore, supports the knowledge that the bacteriological spectrum is necessary to start prompt and targeted antibiotic therapy emphasizing the diagnosis picture suggestive of infection.

The main objective of this study was to analyze the results of the bacteriological examinations collected from the wounds of the patients at admission to the hospital, different to most of the studies found in the literature which limited the correlation of data obtained with those existing in the scientific area of research.

In the analyzed samples, Gram-positive cocci were isolated in 75.85% of the cases, of which staphylococcus germs represented 64.7%. The most frequently isolated species were coagulase-negative Staphylococci (CoNS) at 33.37% and *S. aureus* at 14.14%**,** unlike other studies in which *S. aureus* strains were predominant, followed by CoNS [14,15]. These Staphylococci species are known as part of the habitual integumental microbacterial flora, as opportunistic germs which can become pathogenic agents in cutaneous and wound infections, bone and joint septic determinations, infections of the urogenital, respiratory, and digestive system, and even in endocarditis and hematogenous dissemination, with septicemia, if the integumental/mucous continuity is interrupted. The evolution to infection depends on the dose and virulence of the germs that invade the tissues, as well as on the defense mechanisms of the macro-organism; therefore, it is clear why infections caused by these germs are more frequent in patients with weakened immune systems. The distinction between infection and contamination in the case of these species is still an important issue, both from the perspective of acquiring antibiotic resistance and by introducing them into surgical wound infections, where CoNS and *S. aureus* frequently appear. Concerning CoNS, it must be considered that these germs can be considered a reservoir of resistance plasmids (R plasmids) that can be transferred to *S. aureus* and other species of Gram-positive cocci [15,16,17,18].

Of the staphylococci identified, *S. aureus* MRSA is one of the most important pathogenic agents incriminated in surgical wound infections. Despite the efforts made globally to limit the infections caused by this pathogenic agent, its occurrence frequency is higher at the community level. This aspect is very important because the colonizing strain can cause local or systemic infections in certain cases (it is estimated that approximately 15–45% of patients colonized with MRSA will develop an infection with this pathogenic agent in the following period), in which the patients with MRSA infectons have up to 64% higher risk of death than the persons infected with sensitive strains [19].

We identified 37 cases (3.59% of total strains) in this study contaminated–colonized with MRSA at hospitalization, which we considered to fall within the situations with high risk of infection evolution, given the interruption of integumental continuity after the mechanical trauma (twenty-one cases), thermal trauma (nine cases), and infections of soft, bone and joint tissues (seven cases).

In the context of an existing gateway of germs and decreased body reactivity in the most studied pathological entities (burns, traumas with tissue damage, infections of soft, bone, and joint tissues), we considered that the follow-up of the clinical and paraclinical elements’ evolution of the studied patients, whose bacteriological results showed the presence of Staphylococci species at admission, was also important. The clinical follow-up proved to be necessary in light of the information provided by the scientific literature on the high frequency of Staphylococci in the etiopathogeny of the wound and soft tissue infections [19,20,21].

Concerning the Gram-negative bacilli, 259 strains (25.15%) were identified, the most frequent being *Klebsiella* spp. and *P. aeruginosa* species, in agreement with the literature [22,23,24,25].

The Gram-negative strains producing ESBL (Extended-Spectrum Beta-Lactamase) were isolated in a proportion of 1.35%, the most frequent being *Klebsiella* spp. and *E. coli*, which correlated with other studies. The increasing emergence of these bacterial strains has become a major medical issue because their presence is associated with severe infections (often with evolution to exitus), and they are difficult to treat due to the concerning spectrum of antibiotic resistance (from varied classes). It must be also mentioned that the resistance mechanism of these strains can be transferred from one bacterium to another, so the issue of bacterial resistance has broad implications [17,26,27].

In agreement with the recommendations found in the literature, in the case of patients identified with positive cultures of the above mentioned multiresistant strains (producing ESBL and MRSA), high precaution measures were taken along with the standard usual measures of nosocomial infection prevention, represented by isolating or grouping into separate wards those patients (in the limiting context of existing administrative possibilities) to avoid the contamination of other patients and medical staff [28].

As a specificity of the Romanian Public Health System, the phenomenon of professional liability related to HAIs is not so frequent as in the developed European countries [29]. In this context, the occurrence of a complication causally related to the development of an infection contracted during a hospital stay can lead extremely rarely to compensation for the injured patients. This is the reason why the number of judgments regarding HAIs drawn up by the Civil Courts in Romania has been insignificant in recent years. However, due to the inter-dependency of the length of stay and the incidence of hospital acquired infection [30], it has become important to assess the influence of HAIs on the increasing healthcare costs [31]. For example, we could ascertain that, in the studied time period, the incidence of hospital acquired infection increased the length of stay in EUHBC by 4.2 days (from 5.15 to 9.35 days) and the care cost of a patient by 82% (represented by a supplementary amount of EUR 1449 over the EUR 1770 spent as medium cost by stay). In Romania, the entire amount of the hospital stay cost is attributable to the National Public Insurance System.

Finally, the most relevant observation in **our** opinion is that the main role of the hospital/hospital environment in “infecting” hospitalized patients can be demythologized based on objective criteria following the results obtained. The results of the research show that, to the same extent, not only does the hospital environment infect admitted patients with multiresistant germs (as nosocomial infections), but also the patients are continuously infecting the hospital by importing at the moment of admission the community’s multidrug-resistant bacteria, which colonize them.

## 6. Conclusions

1. Wounds that occurred due to trauma, or older infected wounds, are frequently contaminated at admission to the hospital with bacteria coming from the adjacent integument or the external environment (lately even multiresistant bacteria).
2. The most frequently isolated microorganisms from the wounds at admission were Coagulase-negative Staphylococci, *S. aureus*, *Staphylococcus* spp., *Klebsiella* spp., *Enterococcus* spp., and *P. aeruginosa*.
3. Among the multiresistant bacteria isolated at admission, 4.75% were identified: *S. aureus* (MRSA), *Enterococcus* spp. (HLAR), and strains producing extended-spectrum beta-lactamases: *Klebsiella* spp., *E. coli*, and *Serratia* spp.
4. The most frequent bacterial associations encountered in multimicrobial cultures were CoNS-*Enterococcus* spp., CoNS-*S. aureus*, CoNS-*Klebsiella* spp., and other combinations of the four mentioned above.
5. Microorganisms circulating in the Plastic Surgery ward upon the hospitalization of patients with septic potential pathology are not only subject to the multiresistance phenomenon, but they are also a part of it; thus, understanding the antibiotic resistance spectrum can lead to stopping the administration of the usual antibiotics as empirical therapy (inefficient in these conditions, and even possibly responsible for amplifying the multiresistance phenomenon).
6. The presence of multiresistant community strains that were isolated from wounds at the time of hospitalization requires the administration of antimicrobial agents based on the antibiotic sensitivity test.
7. The reduction in the microbial resistance to antibiotics requires the consolidation of an epidemiological supervision system of antibiotic resistance for each ward and medical institution.
8. A concerning phenomenon resulting from the study was the emphasis of multiresistant microorganisms prior to admission that could infect or contaminate the patients within the medical system.
9. In the case of pathogenic microorganisms (especially multiresistant ones), the community environment has a larger influence on the medical environment, different to several years ago, when it was unanimously accepted that HAI “altered” the bacteriological configuration of the extra-hospital environment. In this context, the hospital has changed in the present from the aggressor environment to a microbiologically aggressed environment. Despite this aggression, the hospital can and must be an entity that filters and annihilates the phenomenon of microorganism re-entrance to the extra-hospital environment by establishing and monitoring well-documented therapeutic protocols.
10. The results of this study highlight the necessity of a modern approach to HAI management, which, alongside the preventive component, must also include the predictive and personalized ones.
11. The bacteriological status of the patients at admission will not only predict the type of wound infection, but will also be able to ensure their therapeutic management by providing a constant supply with specific antibiotics for the most frequent germs found in each local surgical ward, and establishing adapted protocols and strategies for dealing with the increasing MDR (multiple drug resistance)/XDR (extreme drug resistance) global threat.
12. We strongly consider that the management of any hospitalized surgical trauma case at the present time should include, systematically, the new “personalized” component in order to achieve the best treatment, with particular clinical decisions based on the individual characteristics of each patient.

## Figures and Tables

**Table 6:** Frequently identified bi-microbial associations.

MICROORGANISM	CASE NO.
CoNS-*Enterococcus* spp.	34
CoNS-*S. aureus* (MSSA)	20
CoNS-*Klebsiella* spp.	20
*Klebsiella* spp.-*Acinetobacter* spp.	19
CoNS-*P. aeruginosa*	18
*S. aureus* (MSSA)-*Klebsiella* spp.	18
*Klebsiella* spp.-*Enterococcus* spp.	17
*S. aureus* (MSSA)-*Enterococcus* spp.	16
CoNS-*E. coli*	15
CoNS-*Acinetobacter* spp.	12
*Staphylococcus* spp.-*Enterococcus* spp.	12
*Klebsiella* spp.-*Proteus* spp.	12
CoNS-*Proteus* spp.	11
CoNS-*Staphylococcus* spp.	10
*Klebsiella* spp.-*P. aeruginosa*	10
*Proteus* spp.-*E. coli*	10

**Table 7:** Frequently identified tri-microbial associations.

MICROORGANISM	CASE NO.
*S. aureus* (MSSA)-*Klebsiella* spp.-*Enterococcus* spp.	7
CoNS-*Klebsiella* spp.-*Acinetobacter* spp.	5
*Klebsiella* spp.-*Acinetobacter* spp.-*E. Coli*	5
CoNS-*S. aureus* (MSSA)- *Enterococcus* spp.	4
CoNS-*Klebsiella* spp.-*Proteus* spp.	4
CoNS-*Klebsiella* spp.-*E. Coli*	4
CoNS-*Proteus* spp.-*Enterococcus* spp.	4
CoNS-*Enterococcus* spp.-*P. Aeruginosa*	4
*Klebsiella* spp.-*Acinetobacter* spp.-*P. Aeruginosa*	4
*Klebsiella* spp.-*Acinetobacter* spp.-*Proteus* spp.	4
*Klebsiella* spp.-*Proteus* spp.-*E. coli*	4
CoNS-*Klebsiella* spp.-*Enterococcus* spp.	3
CoNS-*Enterococcus* spp.-*E. Coli*	3
CoNS-*P. Aeruginosa*-*E. Coli*	3
*S. aureus* (MSSA)-*Klebsiella* spp.-*Acinetobacter* spp.	3
*Staphylococcus* spp.-*Klebsiella* spp.-*Enterococcus* spp.	3
*Klebsiella* spp.-*Enterococcus* spp.-*E. Coli*	3
*Klebsiella* spp.-*Enterococcus* spp.-*P. Aeruginosa*	3
*Klebsiella* spp.-*P. Aeruginosa*-*E. Coli*	3

**Table 8:** Frequently identified quadri- (a) and hepta-microbial (b) associations.

**MICROORGANISM (a)**	**Case no.**
CoNS-*Proteus* spp.- *Enterococcus* spp.- *E. Coli*	2
CoNS-*Klebsiella* spp.- *Acinetobacter* spp.- *Proteus* spp.	2
CoNS-*Klebsiella* spp.- *P. Aeruginosa*-*E. Coli*	2
*S. Aureus* (MSSA)-*Klebsiella* spp.- *Entercoccus* spp.- *P. aeruginosa*	2
**MICROORGANISM (b)**	**Case no.**
*S. Aureus* (MSSA)-*Klebsiella* spp.- *Acinetobacter* spp.- *Enterococcus* spp.- *P. Aeruginosa*-*E. Coli*-*Streptococcus* spp.	1

**Table 9:** Antibiotic resistance of the methicillin-resistant Staphylococcus aureus (MRSA) strains isolated from hospitalization samples (* no. of isolated strains).

Penicillin	R	R	R	R	R	R	R	R	R	R	R	R	R	R	R	R	R	R	R
Oxacillin	R	R	R	R	R	R	R	R	R	R	R	R	R	R	R	R	R	R	R
Erythromycin	R	R	R	R	R	R	R	R	R	R	R	R	R	R	S	S	S	S	S
Clindamycin	R	R	R	R	R	R	R	R	R	R	R	R	R	R	S	S	S	S	S
Amikacin	R	R	S	S	S	-	S	S	S	S	-	S	S	S	-	-	S	S	S
Ciprofloxacin	R	R	R	S	R	I	-	-	S	S	S	S	S	S	S	S	S	S	S
Gentamicin	R	R	R	R	S	S	S	S	S	S	S	-	S	S	S	S	S	S	S
Levofloxacin	R	R	-	-	-	-	S	S	-	-	-	S	S	S	-	-	S	-	S
Chloramphenicol	S	S	S	S	S	S	R	S	S	S	S	S	S	S	R	S	S	S	S
Trimethoprim/sulfamethoxazole	-	-	R	S	S	S	S	S	S	S	S	S	S	S	S	S	-	S	S
Rifampicin	R	R	S	S	S	S	S	S	S	-	S	S	-	S	S	S	S	S	S
Linezolid	R	S	S	S	S	S	S	S	S	S	S	S	S	S	S	S	S	S	S
Teicoplanin	R	S	S	S	S	S	S	S	S	S	S	S	S	S	S	-	S	S	S
	1 *	1 *	1 *	1 *	1 *	1 *	1 *	1 *	11 *	1 *	5 *	1 *	1 *	1 *	1 *	1 *	2 *	4 *	1 *

**Table 10:** Antibiotic resistance of the Gram-negative producing extended-spectrum beta-lactamases (ESBL) and *Enterococcus* spp. (HLAR) strains, isolated from hospitalization samples.

						ESBL											HLAR	
		*Klebsiella* spp.														*Enterococcus* spp.		
								*E. coli*						*Serratia* spp.				
**Ampicillin**	R	R	R	R	R		R	R			R			**-**		S	S	S
**Amoxicillin/**	S	R	I	R	R		S	I			S			-		-	-	-
**clavulanic acid**																		
**Amikacin**	S	S	S	S	S		R	S			S			-		-	-	-
**Ceftazidime**	R	R	I	I	S		R	R			R			-		-	-	-
**Ciprofloxacin**	R	S	S	S	S		S	S			S			S		R	R	R
**Ceftriaxone**	R	R	R	S	S		R	R			R			-		-	-	-
**Chloramphenicol**	R	S	R	S	S		S	S			S			S		S	R	R
**Clindamycin**	-	-	-	-	-		-	-			-			S		-	-	-
**Erythromycin**	-	-	-	-	-		-	-			-			S		R	R	R
**Imipenem**	S	S	S	S	S		S	S			S			-		-	-	-
**Levofloxacin**	-	S	-	S	S		-	-			-			S		R	R	R
**Linezolid**	-	-	-	-	-		-	-			-			S		S	S	S
**Meropenem**	-	S	-	S	S		-	-			-			-		-	-	-
**Oxacillin**	-	-	-	-	-		-	-			-			S		-	-	-
**Penicillin**	-	-	-	-	-		-	-			-			R		S	S	S
**Rifampicin**	-	-	-	-	-		-	-			-			-		R	I	S
**Teicoplanin**	-	-	-	-	-		-	-			-			S		S	S	S
**Trimethoprim/sulfamethoxazole**	S	S	S	S	S		-	R			-			-		-	-	-
**Vancomicin**	-	-	-	-	-		-	-			-			-		S	S	S

**Table 11:** Dynamic distribution of isolated strains.

	Admission Sample	Intermediary Sample	Final Sample
CoNS	344	43	30
*S. aureus* (MRSA)	41	14	13
*S. aureus* (MSSA)	150	17	7
*Staphylococcus* spp.	132	16	14
*Klebsiella* spp.	79	9	4
*Klebsiella* spp. (ESBL)	5	3	1
*Acinetobacter* spp.	38	7	9
*Proteus* spp.	30	5	2
*Enterococcus* spp.	78	21	7
*Enterococcus* spp. (HLAR)	3	6	3
*Enterobacter* spp.	4	2	1
*Serratia* spp.	2	1	1
*Serratia* spp. (ESBL)	1	0	0
*P. aeruginosa*	58	18	13
*E. coli*	39	9	3
*E. coli* (ESBL)	3	2	1
*Streptococcus* spp.	23	4	3
*Candida* spp.	1	3	2
*Proteus* spp. (ESBL)	0	1	0
*Chryseobacterium indologenes*	0	1	0
	1031	182	114

## Data Availability

Available on reasonable request.
